# Longitudinal Physical Function Trajectories In Older Adults With Mental Illness

**DOI:** 10.1093/geroni/igaf122.4298

**Published:** 2025-12-31

**Authors:** Yuelin Li, Yuelong Xu, Zhirui Deng, Rezvaneh Manzour, Kyeongra Yang, Yue Coco Dong, Heeyoung Lee

**Affiliations:** University of PIttsburgh, Pittsburgh, Macau; University of Pittsburgh, Pittsburgh, Pennsylvania, United States; University of Pittsburgh, Pittsburgh, Pennsylvania, United States; University of Pittsburgh, Pittsburgh, Pennsylvania, United States; Rutgers University, Newark, New Jersey, United States; University of Pittsburgh, Pittsburgh, Pennsylvania, United States; University of Pittsburgh, Pittsburgh, Pennsylvania, United States

## Abstract

Mental illness is recognized as a risk factor for accelerated physical aging and earlier onset of morbidity and mortality, yet its impact on functional outcomes in aging remains underexplored. This study examined longitudinal changes in physical functioning by mental illness using data from 13,419 adults aged ≥50 in the U.S. Health and Retirement Study (2006–2018). Mental illness was defined by the presence of depressive symptoms and/or psychiatric history, categorized as General (neither), Depression-only, Either, or Both. Physical functioning was assessed with grip strength, balance, and walking speed. Generalized estimating equations modeled longitudinal trajectories. Of the sample, 50.7% were in the General group, 20.9% in Depression-only, 23.7% in Either, and 4.7% in Both. At baseline, adults in the Both group exhibited poorer physical functioning than the General group, including lower grip strength (31.7 vs. 34.7 kg), slower walking speed (3.39 vs. 3.12 seconds), and lower balance test completion (28.5 vs. 35.1; ps < .001). Over time, all groups experienced functional decline, but walking speed declined more rapidly in the Both group (from 3.39 to 3.58 seconds) compared with the General group (p < .05). Baseline differences in grip strength and balance persisted over time with comparable rates of decline. Findings provide evidence that mental illness is a determinant of functional aging, with physical functioning serving as a measurable phenotype. Results underscore the need to integrate mental and physical health approaches in research and practice to reduce disparities and promote healthy aging among people with mental illness.

